# Regulation of laryngeal squamous cell cancer progression by the lncRNA RP11‐159K7.2/miR‐206/DNMT3A axis

**DOI:** 10.1111/jcmm.15331

**Published:** 2020-05-04

**Authors:** Xin Wang, Boyu Yu, Qianqian Jin, Junyi Zhang, Bingrui Yan, Like Yang, Yushan Li, Qiuying Li, Peng Wang, Chuanhui Sun, Ming Liu, Linli Tian, Yanan Sun

**Affiliations:** ^1^ Department of Otorhinolaryngology, Head and Neck Surgery The Second Affiliated Hospital Harbin Medical University Harbin China; ^2^ Department of Otorhinolaryngology, Head and Neck Surgery Puyang Oilfield General Hospital Puyang China; ^3^ Department of Otorhinolaryngology Daqing Oilfield General Hospital Daqing China; ^4^ Department of Otolaryngology Daqing First Hospital Daqing China; ^5^ Department of Otorhinolaryngology, Head and Neck Surgery The First Affiliated Hospital Guizhou University of Traditional Chinese Medicine Guizhou China

**Keywords:** CRISPR/Cas9, DNMT3A, laryngeal squamous cell cancer (LSCC), long non‐coding RNA, microRNA

## Abstract

Long non‐coding RNAs (lncRNAs), which are longer than 200 nt, have been proved to play a role in promoting or inhibiting cancer progression. The following study investigated the role and underlying mechanisms of lncRNA RP11‐159K7.2 in laryngeal squamous cell carcinoma (LSCC) progression. Briefly, in situ hybridization (ISH) and real‐time quantitative PCR (RT‐qPCR) showed higher expression of RP11‐159K7.2 in LSCC tissues and cell lines. Patients with low expression level of RP11‐159K7.2 lived longer compared to those with high expression of RP11‐159K7.2 (*χ* ^2^ = 39.111, ***P < 0.001). Multivariate Cox regression analysis suggested that lncRNA RP11‐159K7.2 was an independent prognostic factor for LSCC patients (HR = 2.961, ***P < 0.001). Furthermore, to investigate the potential involvement of RP11‐159K7.2 in the development of LSCC, we knocked out the expression of endogenous RP11‐159K7.2 in TU‐212 cells and AMC‐HN‐8 cells via CRISPR/Cas9 double vector lentiviral system. RP11‐159K7.2 knockout decreased LSCC cell growth and invasion both in vitro and in vivo. Mechanically, we found that RP11‐159K7.2 could positively regulate the expression of DNMT3A by sponging miR‐206. In addition, a feedback loop was also discovered between DNMT3A and miR‐206. To sum up, these findings suggest that lncRNA RP11‐159K7.2 could be used as a potential biomarker for prognosis and treatment of LSCC.

## 1 INTRODUCTION

Laryngeal cancer, more than 95% of which are squamous cell carcinomas (SCC), is the second most common malignant neoplasm in head and neck. Its incidence has remarkably increased over the recent years due to the high smoking rates, industrialization and ageing.^1^, ^2^ Despite considerable progress in surgical techniques, as well as chemotherapy and radiotherapy, the prognosis of advanced laryngeal cancer remains poor.^3^, ^4^, ^5^ Also, the exact molecular mechanisms underlying the carcinogenesis or progression of LSCC remain poorly understood.^6^ The lack of valuable biomarkers and the recurrence and metastasis are the significant problems in LSCC diagnosis and treatment. Therefore, identification of new, sensitive and specific LSCC biomarkers is urgent for the early diagnosis and prognostic evaluation LSCC patients. In addition, it is crucial to explore the molecular mechanisms leading to the pathogenesis of LSCC and discover effective therapeutic targets for suppression of the progression.

Long non‐coding RNA (lncRNA) is non‐coding, with more than 200 nucleotides in length that have an essential role in imprinting, epigenetic regulation, transcriptional and translational regulation.^7^, ^8^ LncRNAs were primitively thought to be the ‘dark matter’ or ‘noise’ of genomic transcription and to be dysfunctional.^9^, ^10^ With the rapid development of molecular biology, accumulating evidence has revealed that lncRNAs are involved in almost every aspect of cellular processes such as cell proliferation, apoptosis and migration.^11^, ^12^ In recent years, an increased understanding of lncRNAs has revealed that they function as potent oncogenes or tumour suppressors in various cancer types.^13^, ^14^ The aberrant expression of lncRNAs has been identified to play a key role in the tumorigenesis and development of laryngeal cancer.^15^, ^16^ Our previous studies also revealed that lncRNA HOTAIR, LET and NEAT1 participate in LSCC progression.^17^, ^18^, ^19^ Nevertheless, the overall pathophysiological contribution of lncRNAs to LSCC needs to be further investigated. Over recent years, ceRNA effect has become a research hotspot.^20^ LncRNAs can mutually regulate one another by competitively binding to microRNAs as long as they share common microRNA‐binding sites. Previous studies have reported the effects of ceRNAs in the lncRNA‐microRNA‐mRNA regulatory network in LSCC.^21^, ^22^ Zhao et al have elucidated the complex regulated mechanism in LSCC via network construction approach.^23^ With microarray analysis, our preliminary experiments showed that RP11‐159K7.2 level was significantly increased in LSCC tissues (data not shown). In the present study, we explored the role and underlying mechanism of a novel lncRNA, RP11‐159K7.2 in LSCC progression. Results showed that RP11‐159K7.2 was overexpressed in LSCC and that RP11‐159K7.2 levels were significantly correlated with cervical lymph node metastasis and prognosis of LSCC patients. Furthermore, we found that down‐regulation of RP11‐159K7.2 could suppress LSCC cell proliferation and invasion both in vitro and in vivo. Moreover, RP11‐159K7.2 was evaluated as a molecular sponge for miR‐206, an additional regulator of DNMT3A expression. Additionally, a feedback loop was also discovered between DNMT3A and miR‐206. We first proposed the important molecular marker role of RP11‐159K7.2 in laryngeal cancer and for the first time demonstrated that it is involved in the regulation of DNA methylation in laryngeal cancer. No RP11‐159K7.2 research report has been seen so far.

## 2 METHODS

### 2.1 Tissue samples

A total of 311 pairs of LSCC and adjacent non‐tumorous tissues were collected from patients undergoing partial or total laryngectomy in the Department of Otorhinolaryngology, the Second Affiliated Hospital of Harbin Medical University, from 2010 to 2013. Patients diagnosed as LSCC had never received any therapy before surgery. Paraffin‐embedded tissues and clinical records of 225 consecutive LSCC patients were retrieved for ISH. Besides, we also collected LSCC tissues and adjacent non‐tumorous tissues from another 86 patients to analyse the levels of RP11‐159K7.2 by RT‐qPCR. The Ethics Committee of Harbin Medical University has approved this study.

### 2.2 RT‐qPCR

Total RNA was extracted from LSCC tissues and cell lines by utilizing TRIzol reagent (Invitrogen, Carlsbad, CA, USA) agreeing to the manufacturer's directions. For mRNA and lncRNA, real‐time PCR was carried out utilizing Power SYBR Green RT‐qPCR Reagents (Applied Biosystems, Foster City, CA, USA). Data were normalized to the GAPDH expression level and further normalized to the negative control. For the detection of microRNA expression, the miR‐206 level was carried out utilizing TaqMan MicroRNA Assays Kit (Applied Biosystems, Carlsbad, CA, USA) agreeing to the manufacturer's directions. Data were normalized to the U6 snoRNA expression level and calculated using the 2^−ΔΔCt^ method. Sequences of the primers are listed in Table 1.

**TABLE 1:** Primers used in the study

Gene	Sequences
RP11‐159K7.2 forward	5′‐GGCACAAGCAAATTACACCA‐3′
RP11‐159K7.2 reverse	5′‐CCAGCAGGGCTAGTTGTTTC‐3′
miR‐206 forward	5′‐GCCCGCTGGAATGTAAGGAAGT‐3′
miR‐206 reverse	5′‐CCAGTGCAGGGTCCGAGGT‐3′
DNMT3A forward	5′‐TATTGATGAGCGCACAAGAGAGC‐3′
DNMT3A reverse	5′‐GGGTGTTCCAGGGTAACATTGAG‐3′
GAPDH forward	5′‐TGAACGGGAAGCTCACTGG‐3′
GAPDH reverse	5′‐TCCACCACCCTGTTGCTGTA‐3′
U6 forward	5′‐GCTTCGGCAGCACATATACTAAAAT‐3′
U6 reverse	5′‐CGCTTCACGAATTTGCGTGTCAT‐3′

Abbreviation: RP11‐159K7.2, long non‐coding RNA RP11‐159K7.2; miR‐206, microRNA‐206; DNMT3A, DNA methyltransferase 3A; GAPDH, glyceraldehyde‐3‐phosphate dehydrogenase; U6, U6 small nuclear RNA.

### 2.3 ISH

In situ hybridization was performed to the 225 paraffin‐embedded samples. Each sample was examined separately and scored by two pathologists. ISH was performed with the RNA scope^®^ 2.5 Assay and HybEZ^TM^ Hybridization System (ACD, Inc 110 VAC). LncRNA RP11‐159K7.2 target, positive and negative control probes were purchased from ACD. Technical and slide quality control have been verified using positive control probe against the common housekeeping protein, peptidyl prolyl isomerase B (PPIB) and a negative control probe targeting the bacterial protein dihydrodipicolinate, reductase (DapB). On a scale from 0 to 3, the same pathologist valued semi‐quantitatively the level of inflammation on microscopic segments, 0: none; 1: <10%; 2: 10%‐50%; and 3: >50%. A score of 2 was used to distinguish between low (<2) and high (≥2) level of RP11‐159K7.2 expression.

### 2.4 Cell culture and construction of LentiCRISPR/Cas9 system

Human LSCC cells (TU‐212 cells, AMC‐HN‐8 cells) and HEK‐293T cells were maintained in Dulbecco's modified Eagle's medium (DMEM) supplemented with 10% foetal bovine serum in a humidified incubator at 37°C with 5% CO_2_. Lenti‐Cas9‐puro, lentiviral vector stably expressing Cas9 which is resistant to puromycin, was constructed and transfected into LSCC cells. Therefore, we established two laryngeal cancer cell lines stably expressing Cas9. Genechem Co., Ltd. (Shanghai, China) engineered and produced 3 single guide RNAs (sgRNAs) targeting human RP11‐159K7.2 gene and marked lentivirus‐sgRNA‐EGFP with enhanced green fluorescent protein (EGFP). Lentivirus‐cas9‐puro vector and three lentivirus‐sgRNA‐EGFP vectors were constructed by Genechem (Shanghai, China). Finally, the sgRNA with the highest efficiency of knockout RP11‐159K7.2 was selected. The sgRNA sequence is as follows: forward: 5′‐TTGCGCTTACTCCTTGCTAA‐3′; reverse: 5′‐CACACAGAGGGATCATGACT‐3′.

### 2.5 MTT assay

To measure cell proliferation after transfection with the Lenti CRISPR/Cas9 system, we performed MTT assay. The LSCC cells (5000 cells) were seeded on 96‐well plates for 48 hours and then incubated in MTT solution (Thermo Scientific, Shanghai, China) for 4 hours. After that, add 100 μL of Formazan solution buffer to each well and mixed thoroughly and then incubate the microplate at 37°C for 4‐18 hours in a humidified incubator. Using the Thermo Plate microplate reader (Rayto Life and Analytical Science Co. Ltd., Germany), samples absorbance at 570 nm was evaluated.

### 2.6 Colony formation assay

After transfection, TU‐212 and AMC‐HN‐8 cells were seeded at an appropriate density into 6‐well culture plates and maintained in DMEM containing 10% FBS. The colonies were fixed with methanol after 2 weeks and stained at room temperature with crystal violet for 30 minutes. The formed colonies were counted in every well.

### 2.7 Transwell assay

For cell invasion analysis, cells (1 × 10^5^) were added to a transwell chamber's upper compartment. Transwell filters (pore size, 8 μm; Falcon; BD Biosciences) were coated on the lower side with 8 μg/μL Matrigel. The cells placed on a 24‐well plate containing DMEM allowed to migrate at 37°C for 24 hours. The cells from the upper compartment were then removed with a cotton swab, and the cells that migrate to the lower face of the filter were fixed in 3.7% formaldehyde (in PBS) and stained with crystal violet for 10 minutes at room temperature. By evaluating the number of cells in three inserts, cell invasion was quantified.

### 2.8 In vivo experiment

Eighteen BALB/c mice, age 5 to 6 weeks, were purchased from Vital River Laboratories (Beijing, China). For tumour growth study, nine mice were included in each group. Mice in the experimental group (n = 9) were injected with 100 µL AMC‐HN‐8 cells transfected with RP11‐159K7.2 CRISPR/Cas9 lentivirus; mice in the control group (n = 9) received an injection of 100 µL AMC‐HN‐8 cells transfected with control lentivirus. The tumour size was measured once a week, and the tumour volume was determined using the simplified formula of a rotational ellipsoid (length × width^2^ × 0.5). Six weeks after implantation, the xenografts were removed from the mice and weighed. The experimental procedures were approved by the Ethics Review Committee of Harbin Medical University.

### 2.9 Bioinformatics analysis

RNA‐seq data of LSCC patients were obtained from The Cancer Genome Atlas (TCGA) dataset (https://cancergenome.nih.gov/). The miRcode (http://www.mircode.org/) database was used to predict relationships between lncRNA and microRNA. microRNA‐mRNA interactions were predicted using starBase (http://starbase.sysu.edu.cn/index.php). Kaplan‐Meier survival curves of microRNA were drawn using the KMplot program (http://kmplot.com/analysis/). The expression relationship between miR‐206 and DNMT3A was predicted by starBase.

### 2.10 Luciferase reporter assay

The downstream of the firefly luciferase (f‐luc) in the pGL3 plasmid was inserted into 3′UTR of RP11‐159K7.2 containing the miR‐206‐binding site and designated RP11‐159K7.2‐WT. Similarly, the downstream of the firefly luciferase (f‐luc) was inserted into 3′UTR of RP11‐159K7.2 with mismatch mutations in miR‐206 seed complementary site and designated RP11‐159K7.2‐Mut. HEK‐293T cells were cultured in 96‐well plates and cotransfected with 400 ng of either RP11‐159K7.2‐WT, RP11‐159K7.2‐Mut, 50 ng pRL‐TK (Promega, USA) and 50 nmol/L miR‐206 or scramble microRNA negative control (miR‐NC). The pRL‐TK Renilla luciferase plasmid was used as an internal control. Likewise, the miR‐206 or miR‐NC was cotransfected with DNMT3A‐WT or DNMT3A‐Mut into HEK‐293FT cells. Dual‐luciferase reporter assay was used to measure firefly and Renilla luciferase activities 48 hours after transfection. The outcomes have been demonstrated as relative luciferase activity (firefly luciferase/Renilla luciferase).

### 2.11 Western blot analysis

As previously stated, LSCC cells were gathered and analysed using Western blot to evaluate the expression of DNMT3A.^24^ Total protein was extracted using RIPA Lysis Buffer (Beyotime Biotechnology, China). BCA Protein Assay Kit (Beyotime Biotechnology, China) was used to measure the protein concentration. DNMT3A antibody (Abcam, Shanghai, China) was diluted to 1:1000. The expression levels of DNMT3A were expressed as a ratio of the expression of GAPDH.

### 2.12 Immunohistochemistry

Xenografts removed from the mice were fixed in paraformaldehyde and then embedded in paraffin. Endogenous peroxidase activity was blocked in deparaffinated sections. A primary rabbit polyclonal anti‐DNMT3A (1:2000) obtained from Abcam (ab188470) was used; the primary antibody reactions were incubated overnight at 4°C after blocking with 1% bovine serum albumin. Sections were submerged in the biotinylated goat anti‐rabbit secondary antibody for 1 hour on the morrow. After incubation with the VECTSTAIN Elite ABC complex, development was performed with AEC chromogen. Expression was assessed using a composite score obtained by multiplying the value of staining intensity by the percentage of positive cells.

### 2.13 5‐Aza‐dC treatment and Bisulfite sequencing

LSCC cells (5 × 10^3^/well) were seeded on 96‐well plates for 5‐Aza‐2′‐deoxycytidine (5‐Aza‐dC, Sigma, St Louis, MO, USA) treatment and routinely cultured for 24 hours. LSCC cells were treated with 3 μmol/L 5‐aza‐dc for 72 hours, and DMSO was used as the control. For Bisulfite sequencing PCR (BSP), genomic DNA was extracted from TU‐212 and AMC‐HN‐8 cells using the TIANamp Genomic DNA kit (Tiangen Biotech, Beijing, China), and bisulfite conversion was then performed with the EZ DNA Methylation‐Gold Kit (Zymo Research). The primers (forward) 5′‐TTTTATAGATTGTGGGGTAGGTGAT‐3′ and (reverse) 5′‐AAAAAATCCAACAAAAAAATTTTCC‐3′ were used to amplify the DNA sequences of CpG sites in PCR procedure. The amplified fragments were cloned into the pGEMT Easy vector (Promega, Madison, WI, US), and 10 clones were subjected to DNA sequencing.

### 2.14 Statistical analysis

Statistical analyses were performed with SPSS version 17.0 software. The association of RP11‐159K7.2 with clinicopathological features was assayed by *χ* ^2^ test and Student's *t* test. The OS was evaluated by the Kaplan‐Meier method. Log‐rank test was used for survival analysis. Multivariate Cox proportional hazards analysis was performed with survival as the dependent variable. Three levels of significance (*P < 0.05; ** *P* < 0.01; and ***P < 0.001) were used for all the tests.

## 3 RESULTS

### 3.1 LncRNA RP11‐159K7.2 is overexpressed in LSCC tissues and cell lines

**FIGURE 1:**
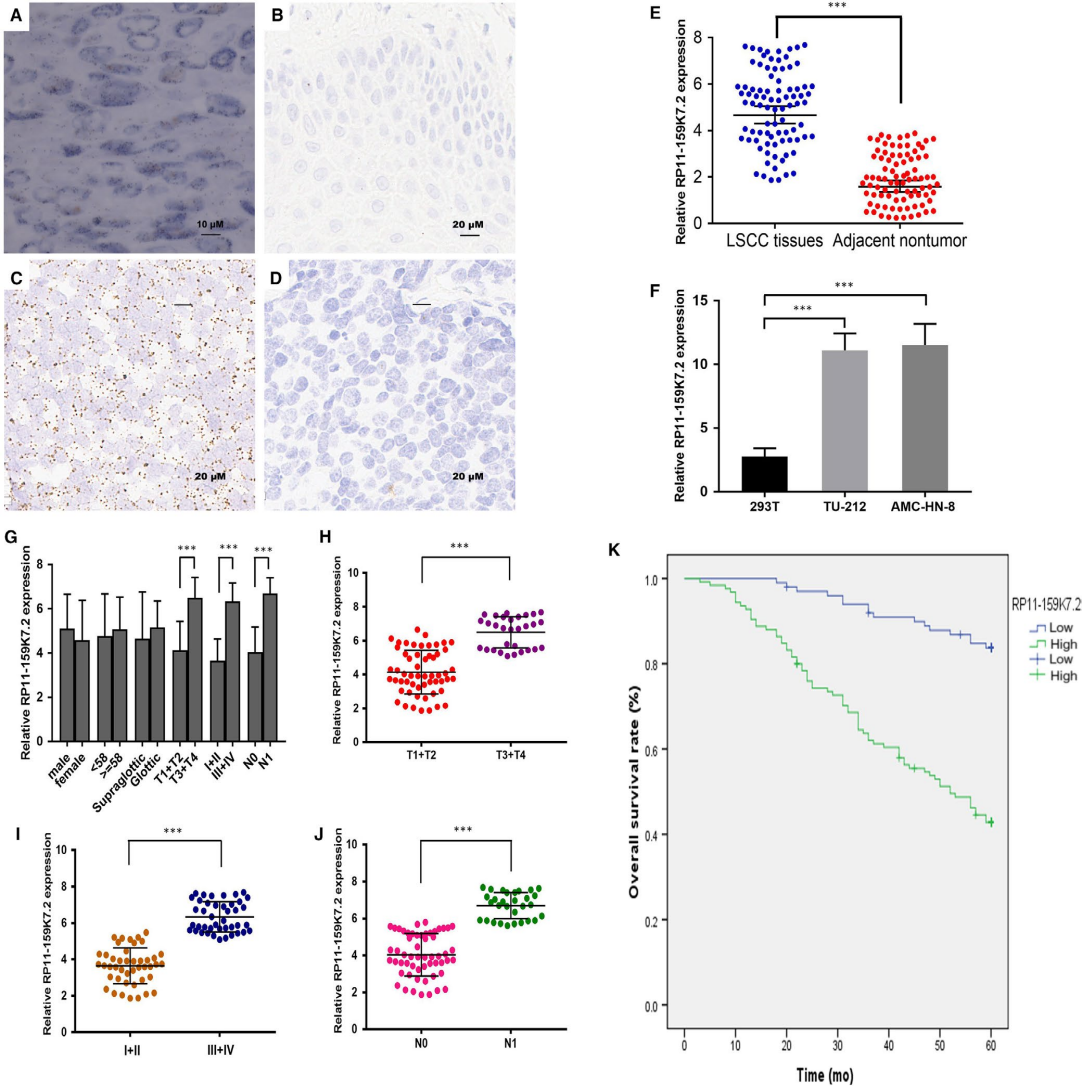
The RP11‐159K7.2 expression level was up‐regulated in LSCC tissues and cell lines. In situ hybridization assay was used to determine the expression of RP11‐159K7.2. A, LSCC tissue; B, adjacent non‐tumorous tissue; C, positive control; D, negative control. E, RT‐qPCR was performed to validate RP11‐159K7.2 expression in 86 pairs of LSCC tissue and adjacent non‐tumorous tissue (***P < 0.001). F, RP11‐159K7.2 expression was higher in LSCC cells compared with a normal cell line (***P < 0.001). G‐J, Tumours with advanced clinical stages, with T3‐4 grade or with lymph node metastasis expressed higher levels of RP11‐159K7.2 ***P < 0.001. K, Kaplan‐Meier survival analysis indicated that high RP11‐159K7.2 expression levels in LSCC were significantly associated with worse OS (***P < 0.001)

In situ hybridization assay was performed to identify the localization of RP11‐159K7.2 in 225 pairs of LSCC and adjacent non‐tumorous tissues. Results suggested RP11‐159K7.2 was located in the cell nuclei and cytoplasm. In addition, high expression of lncRNA RP11‐159K7.2 was detected in cancerous tissues. Moreover, the results from RT‐qPCR revealed that the expression of RP11‐159K7.2 was higher in tumour tissues than that in adjacent non‐tumorous tissues, which was consistent with ISH (Figure 1A‐E). Additionally, RP11‐159K7.2 was observed to be highly expressed in LSCC cell lines TU‐212 and AMC‐HN‐8 compared with HEK‐293T cells using RT‐qPCR (Figure 1F).

### 3.2 Correlations between the expression of RP11‐159K7.2 and clinicopathological parameters

We analysed the correlation between RP11‐159K7.2 expression and the clinicopathological parameters of patients with LSCC. As shown in Table 2, the LSCC patients with high RP11‐159K7.2 expression were more likely to develop tumour (**P = 0.002) and reach higher clinical stage (*P = 0.017). In addition, lymphatic invasion (*P = 0.017) and higher recurrence (*P = 0.014) were observed in patients with high RP11‐159K7.2. However, there were no significant correlations between RP11‐159K7.2 expressions based on age, gender or tumour location. Furthermore, the results of RT‐qPCR in 86 pairs of LSCC tissues showed that the levels of RP11‐159K7.2 were positively associated with tumour classification (***P < 0.001), higher clinical stage (***P < 0.001) and lymphatic metastasis (***P < 0.001) (Figure 1G‐J).

**TABLE 2:** Relationship between RP11‐159K7.2 expression level and clinicopathological parameters of LSCC

Characteristics (n)	RP11‐159K7.2 expression		χ^2^	*P*
	High	Low		
Sex			0.429	0.512
Male (169)	96	73		
Female (56)	29	27		
Age (y)			0.241	0.624
≥58 (139)	79	60		
<58 (86)	46	40		
T classification			9.754	0.002**
T1‐2 (146)	70	76		
T3‐4 (79)	55	24		
Recrudescence			6.036	0.014*
Negative (174)	89	85		
Positive (51)	36	15		
Lymph node metastasis			5.714	0.017*
Negative (162)	82	80		
Positive (63)	43	20		
Primary location			0.023	0.879
Supraglottic (91)	50	41		
Glottic (134)	75	59		
Clinical stage			5.692	0.017*
I‐II (115)	55	60		
III‐IV (110)	70	40		

Note

In situ hybridization was performed to all 225 surgical samples. The same pathologist semi‐quantitatively appreciated the level of inflammation on microscopic sections on a scale from 0 to 3, 0: none; 1: <10%; 2: 10%‐50%; and 3: >50%. A score of 2 was used to distinguish between low (<2) and high (≥2) levels of RP11‐159K7.2 gene expression. Data were analysed by chi‐squared test. *P*‐value with * indicates statistically significant.

### 3.3 High RP11‐159K7.2 expression predicts poor prognosis in LSCC

The association between RP11‐159K7.2 expression and overall survival (OS) of patients with LSCC was evaluated by Kaplan‐Meier analysis and log‐rank test. Kaplan‐Meier survival analysis demonstrated that patients with low RP11‐159K7.2 expression lived longer (*χ* ^2^ = 39.111, ***P < 0.001, Figure 1K). To further explore the association between RP11‐159K7.2 and prognosis, Cox regression analysis was conducted. Univariate analysis showed that tumour stage, clinical stage, lymph node metastasis, recrudescence and RP11‐159K7.2 expression were significantly associated with OS. Cox proportional risk model was used to analyse the risk factors with statistical significance in univariate analysis. Multivariate analysis showed that RP11‐159K7.2 was one of the risk factors for prognosis of LSCC (Table 3). These results demonstrated that RP11‐159K7.2 has an important role in determining the prognosis of LSCC.

**TABLE 3:** Cox univariate and multivariate analysis of prognostic factors in LSCC (n = 225)

Variable for overall survival	Univariate analysis		Multivariate analysis	
	HR (95% CI)	*P*‐value	HR (95% CI)	*P*‐value
Gender	1.738 (0.996‐3.035)	0.052	—	—
Age (y)	0.785 (0.512‐1.201)	0.263	—	—
Primary location	1.409 (0.922‐2.151)	0.113	—	—
T classification	4.776 (3.632‐6.282)	<0.001	2.709 (1.552‐4.728)	<0.001***
Clinical stage	7.694 (5.381‐11.000)	<0.001	2.140 (1.080‐4.242)	0.029*
Lymph node metastasis	5.558 (3.614‐8.548)	<0.001	5.279 (2.794‐9.973)	<0.001***
Recrudescence	3.918 (2.535‐6.055)	<0.001	—	—
RP11‐159K7.2 expression	4.865 (2.822‐8.388)	<0.001	2.961 (1.605‐5.463)	<0.001***

Abbreviations: CI, confidence interval; HR, hazard ratio.

* *P* < 0.05;

** *P* < 0.01; and

*** *P* < 0.001.

### 3.4 RP11‐159K7.2 knockout inhibited the proliferation and invasion of LSCC cells

**FIGURE 2:**
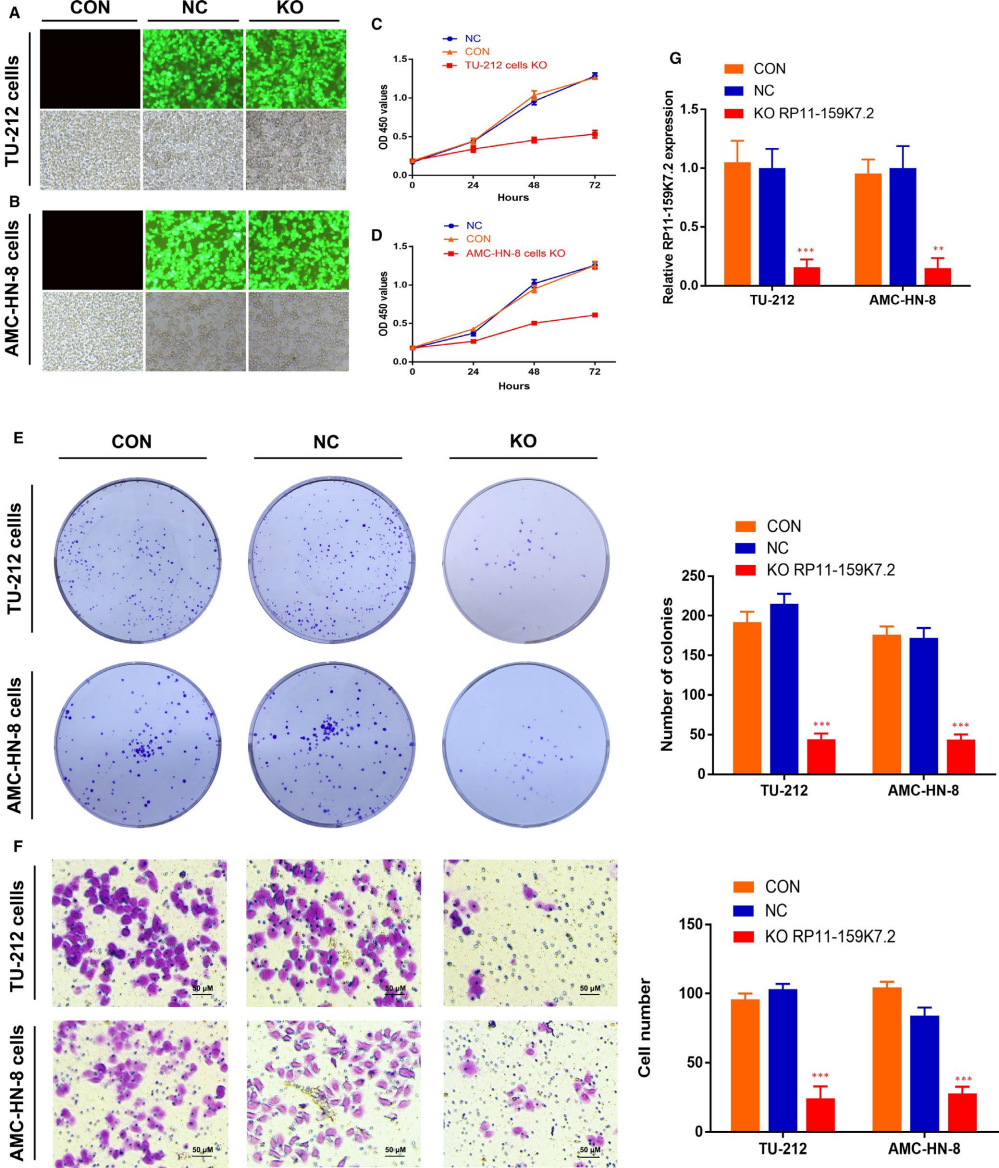
RP11‐159K7.2 inhibition reduces LSCC cell proliferation and invasion. A‐B, The transfection and knockout efficiency were estimated 72 h after transfection with the lentivirus‐sgRNA‐EGFP. Enhanced green fluorescent protein (EGFP) expression in transfected cells was observed by light and fluorescence microscopy, respectively. C‐D, The proliferation of LSCC cells transfected with LentiCRISPR/Cas9 system was decreased at the different time point (24, 48 and 72 h, respectively) compared with the controls. CON, no transfection group; NC, negative control group; KO, RP11‐159K7.2 knockout group. E‐F, The transfection of lentivirus‐sgRNA‐EGFP inhibited the proliferation and invasion of LSCC cells ***P < 0.001. G, Efficiency of RP11‐159K7.2 expression in RP11‐159K7.2 down‐regulated TU‐212, and AMC‐HN‐8 cells were evaluated via RT‐qPCR

To explore the potential involvement of RP11‐159K7.2 in the development of LSCC, we knocked out the expression of endogenous RP11‐159K7.2 in TU‐212 cells and AMC‐HN‐8 cells via CRISPR/Cas9 double vector lentiviral system (Figure 2A,B). MTT assay, colony formation assay and matrigel invasion assay were performed to detect the proliferation and invasion of LSCC cells. RP11‐159K7.2 knockout dramatically inhibited the proliferation of cancer cells after 48 and 72 hours (Figure 2C‐E). Moreover, lower invasion behaviour was observed in RP11‐159K7.2 knockout group compared with the control group (Figure 2F **)**. In addition, the knockout efficiency was verified by using RT‐qPCR (Figure 2G).

### 3.5 RP11‐159K7.2 knockout inhibits the growth of LSCC in vivo

**FIGURE 3:**
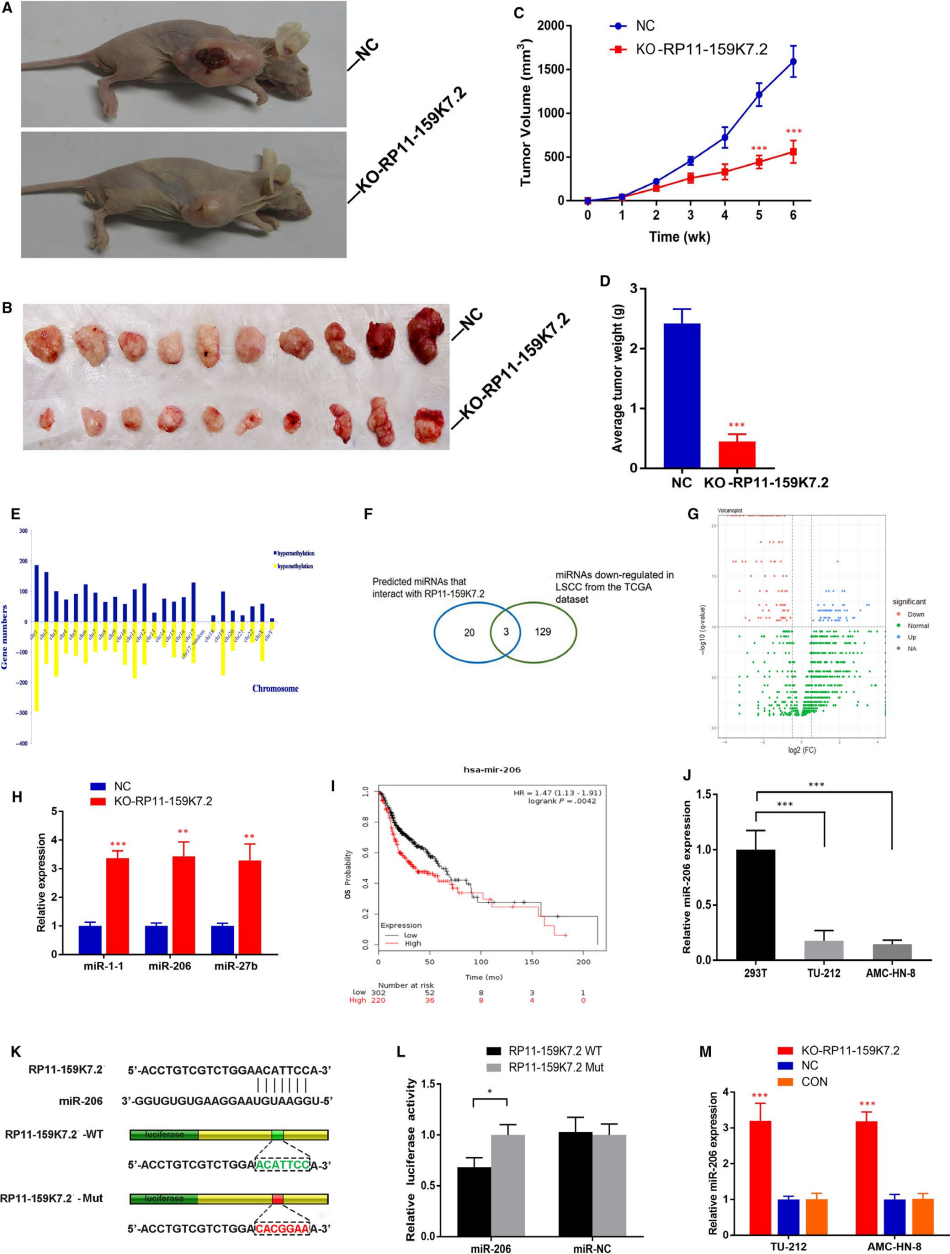
Knockout of RP11‐159K7.2 by CRISPR/Cas9 inhibits LSCC growth in vivo. A, Representative mouse injected with EGFP negative control lentivirus or RP11‐159K7.2 lentivirus‐sgRNA‐EGFP. B, Tumours were dissected after 6 weeks. Tumour volume (C) and tumour weight (D) in RP11‐159K7.2 lentivirus‐sgRNA‐EGFP group were significantly less than in the control group (***P < 0.001). n = 9 mice per group. RP11‐159K7.2 targets miR‐206 to interact with miR‐206 negatively. E, Distribution of differentially methylated regions (DMRs) among different chromosomes after knockout of RP11‐159K7.2. F, Candidate downstream microRNAs of RP11‐159K7.2 predicted by miRcode and the most down‐regulated microRNAs acquired from TCGA, and three microRNAs were screened out. G, microRNAs (with fold change <−0.5 and FDR < 0.1) plotted as a volcano plot. H, RT‐qPCR was performed to determine the relative expression of the three microRNAs in AMC‐HN‐8 cells transfected with RP11‐159K7.2 lentivirus‐sgRNA‐EGFP or EGFP negative control lentivirus. I, Kaplan‐Meier survival curves for miR‐206 associated with overall survival in HNSC. J, miR‐206 was observed to be significantly decreased in LSCC lines (TU‐212, AMC‐HN‐8) compared with the HEK‐293T cell line using RT‐qPCR, normalized to U6 as an endogenous control. K, A schematic diagram showing the binding sites between miR‐206 and RP11‐159K7.2. L, AMC‐HN‐8 cells were transfected with RP11‐159K7.2‐WT or RP11‐159K7.2‐Mut. RP11‐159K7.2‐WT significantly decreased luciferase activity of miR‐206, and there was no significant difference in the activity of miR‐NC *P < .05. M, Relative expression of miR‐206 in AMC‐HN‐8 cells transfected with LentiCRISPR/Cas9 system against RP11‐159K7.2 was decreased compared with the controls, measured using RT‐qPCR

To further investigate the effects of RP11‐159K7.2 on the regulation of AMC‐HN‐8 cells progression in vivo, 18 mice were subcutaneously injected with AMC‐HN‐8 cells. Briefly, all mice developed tumours (Figure 3A,B). However, as expected, tumour carrying the RP11‐159K7.2 lentivirus‐sgRNA‐EGFP had a smaller volume and lower weight compared with the control group (Figure 3C,D).

### 3.6 RP11‐159K7.2 regulates DNMT3A expression through modulating miR‐206

MeDIP and microarray hybridization methods were used to examine the global methylation of gene promoters in LSCC cells. The results showed that down‐regulation of RP11‐159K7.2 decreased the global DNA methylation levels of LSCC cells (Figure 3E). These means the expression of RP11‐159K7.5 affects the methylation level of LSCC cells. Besides, lncRNAs have been reported to function as molecular sponge of microRNAs in cancers.^25^, ^26^ Therefore, we speculated that RP11‐159K7.2 might interact with microRNAs in LSCC to regulate the global methylation level of the genome. We predicted 20 microRNAs that interact with RP11‐159K7.2 using bioinformatics methods (http://www.mircode.org/index.php). Additionally, we analysed microRNAs data with LSCC deposited in the Cancer Genome Atlas (TCGA) database (Figure 3F). A total of 129 microRNAs (log2 fold change <−0.5, FDR < 0.1) differentially down‐regulated in LSCC tissues were screened (Figure 3G), after which the 3 microRNAs were shortlisted. miR‐206, miR‐27b and miR‐1‐1 had binding sites to RP11 separately and were down‐regulated in laryngeal cancer. The obtained results revealed that the expression of these three microRNAs increased after RP11‐159K7.2 knockout (Figure 3H).

**FIGURE 4:**
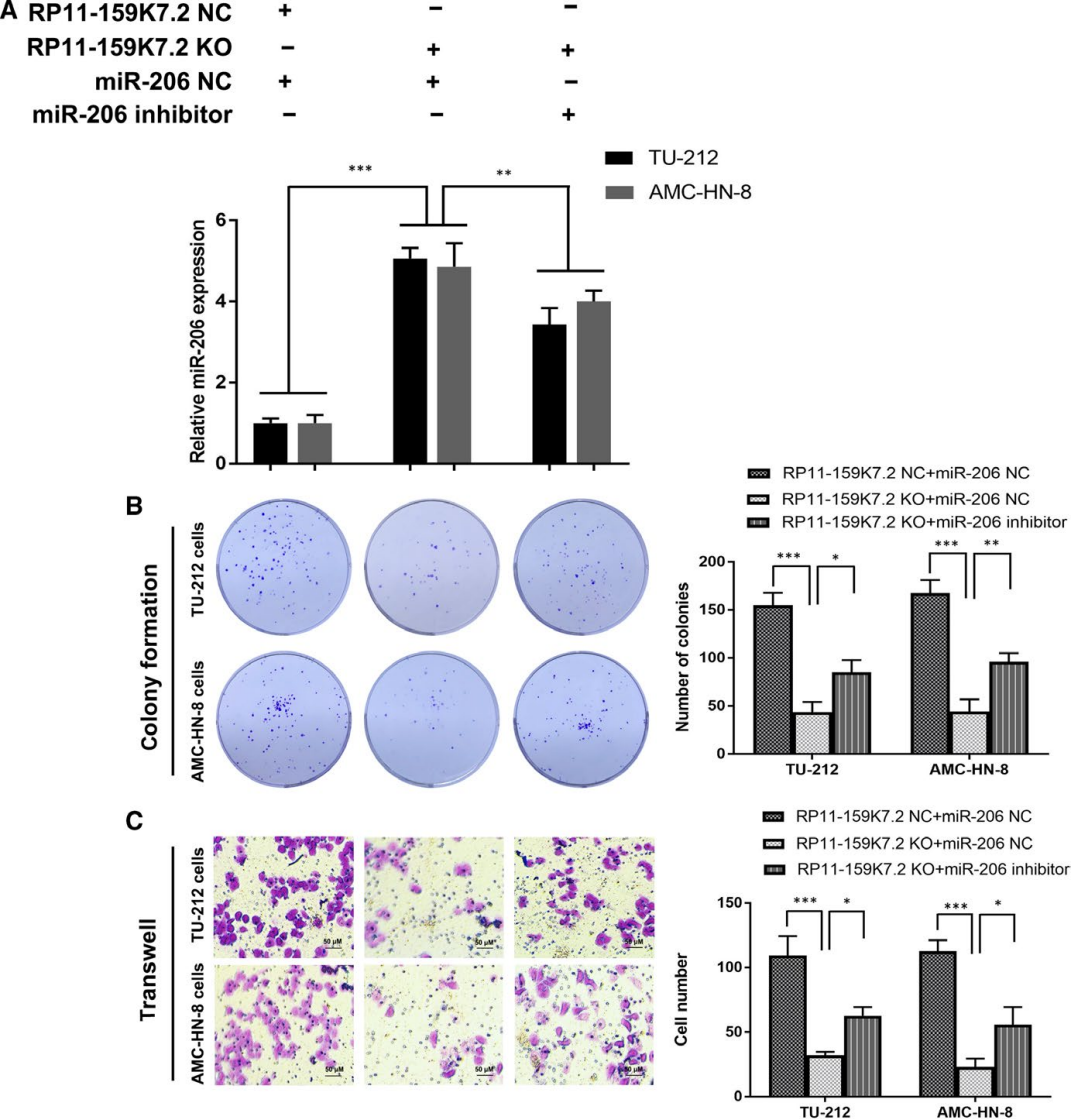
RP11‐159K7.2 expression was suppressed in LSCC cells transfected LentiCRISPR/Cas9 system targeting RP11‐159K7.2, and this could be partly rescued by the cotransfection of miR‐206 inhibitors. A, LSCC cells were transfected with RP11‐159K7.2 NC + miR‐206, RP11‐159K7.2 KO + miR‐NC, RP11‐159K7.2 KO + miR‐206 inhibitor. Colony formation assay (B) and transwell assay (C) showed that miR‐206 inhibitor reversed the inhibition effect of LentiCRISPR/Cas9 system targeting RP11‐159K7.2 on the proliferation and invasion of LSCC cells

We have previously demonstrated that the expression of miR‐206 is decreased in LSCC and that the loss of miR‐206 is important for the proliferation, invasion and apoptosis of LSCC.^27^ Kaplan‐Meier survival curves showed that the overall survival rate in low miR‐206 group patients was poorer than high miR‐206 group patients by bioinformatics analysis of miR‐206 data (Figure 3I). Quantitative real‐time PCR analysis indicated that the expression of miR‐206 was significantly lower in the LSCC cell lines compared to the 293T cells (Figure 3J). Thus, in this study, miR‐206 was selected for further investigation. Next, luciferase reporter gene assay was performed to detect whether miR‐206 specifically targets 3′UTR of RP11‐159K7.2. RP11‐159K7.2‐WT sequence or RP11‐159K7.2‐Mut sequence was cloned into the luciferase reporter vector; RP11‐159K7.2 WT or Mut and miR‐206 or miR‐NC were then transfected into AMC‐HN‐8 cells. The results showed that the luciferase activity in the miR‐206 group was significantly decreased compared with the miR‐NC group, and there was no significant difference in the activity of RP11‐159K7.2‐Mut, suggesting a direct interaction between RP11‐159K7.2 and miR‐206 (Figure 3K‐L). The results of RT‐qPCR further indicated that the knockout of RP11‐159K7.2 significantly increased the miR‐206 expression in TU‐212 and AMC‐HN‐8 cells (Figure 3M), which indicated that miR‐206 was negatively regulated by RP11‐159K7.2. These results were further confirmed by the simultaneous increase in miR‐206 expression when LSCC cells were transfected with LentiCRISPR/Cas9 system targeting RP11‐159K7.2. Colony formation assay and transwell assay showed that miR‐206 inhibitor preserved the inhibition effect of LentiCRISPR/Cas9 system targeting RP11‐159K7.2 on the proliferation and invasion of LSCC cells (Figure 4). Therefore, our data demonstrated that RP11‐159K7.2 promotes cell proliferation and invasion by inhibiting miR‐206.

**FIGURE 5:**
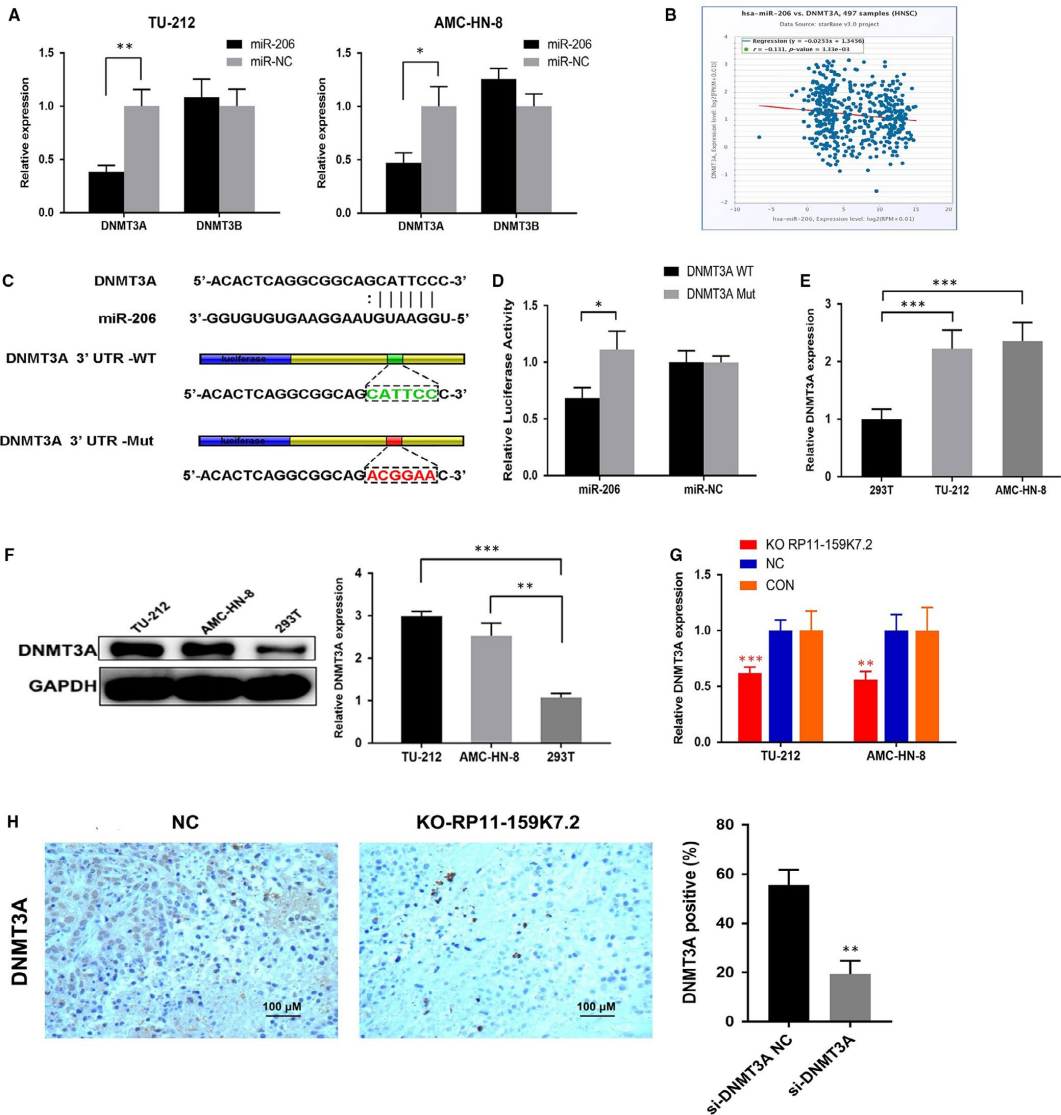
DNMT3A was identified as a direct target of miR‐206. A, Relative expression of the DNMTs in LSCC cells transfected with miR‐206 or miR‐NC measured by RT‐qPCR **P < .01. B, DNMT3A expression was negatively correlated with miR‐206 expression in HNSC. C, Starbase database predicted the target site between DNMT3A and miR‐206. D, AMC‐HN‐8 cells were transfected with DNMT3A‐WT or DNMT3A‐Mut. DNMT3A‐WT significantly decreased luciferase activity of miR‐206, and there was no significant difference in the activity of miR‐NC *P < .05. E, DNMT3A is up‐regulated in LSCC cell lines compared with normal cell line ***P < .001. F, Relative expression of DNMT3A in TU‐212 cell line, AMC‐HN‐8 cell line and HEK‐293T cells was detected by Western blots. G, Relative expression of DNMT3A in AMC‐HN‐8 cells transfected with LentiCRISPR/Cas9 system against RP11‐159K7.2 was decreased compared with the control groups (***P < .001). H, IHC detection of DNMT3A in paraffin‐embedded tissue sections of nude mice xenografts

**FIGURE 6:**
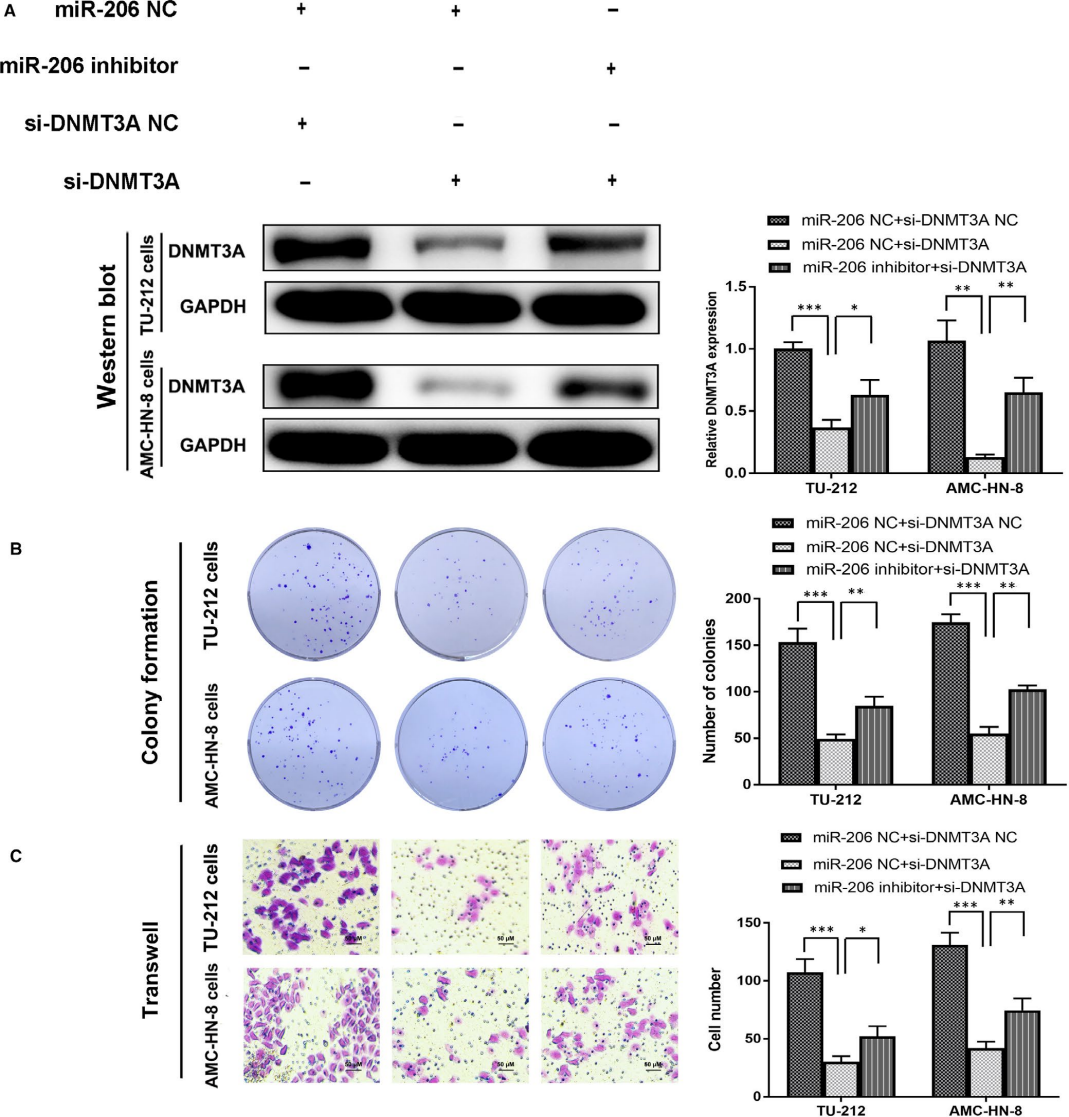
miR‐206 inhibits proliferation and invasion by targeting DNMT3A in LSCC cells. (A) DNMT3A expression was suppressed in LSCC cells transfected siRNA against DNMT3A, and this could be partly rescued by the cotransfection of miR‐206 inhibitors. LSCC cells transfected with siRNA against DNMT3A reduced the ability of proliferation (B) and invasion (C), and the effect could be rescued by the cotransfection of miR‐206 inhibitors as our data suggested that miR‐206 binds to DNMT3A to negatively regulate cell proliferation and invasion

The bioinformatics databases (http://starbase.sysu.edu.cn/index.php) were searched for potential DNMTs that can bind to miR‐206, revealing that DNMT3A and DNMT3B have a putative binding site for the seed sequence of miR‐206. Next, the RNA levels of DNMT3A and DNMT3B were detected by using RT‐qPCR in LSCC cells transfected with miR‐206 and miR‐NC. The results showed that the relative expression decreased was only observed in cells cotransfected with miR‐206 and DNMT3A, but not in DNMT3B in both TU‐212 and AMC‐HN‐8 cell lines (Figure 5A). The correlations between miR‐206 and DNMT3A expression in Head and Neck Squamous Carcinoma (HNSC) suggested that RP11‐159K7.2 may have a critical role in the regulation of DNMT3A expression by modulating miR‐206 in LSCC (Figure 5B). Finally, DNMT3A was chosen over DNMT3B for further analyses. To demonstrate the binding site of miR‐206 in the 3’UTR of DNMT3A, DNMT3A WT or Mut and miR‐206 or miR‐NC were transfected into AMC‐HN‐8 cells. Luciferase reporter assay showed that up‐regulation of miR‐206 could significantly reduce the 3’UTR luciferase activity but could not reduce the luciferase activity of 3′UTR‐MU (Figure 5C,D). Our data indicated that miR‐206 directly modulates DNMT3A by binding the 3′UTR of DNMT3A. Moreover, DNMT3A was highly expressed in LSCC cell lines compared to HEK‐293T cells as proven by RT‐qPCR and Western blotting normalized to GAPDH as endogenous control (Figure 5E,F). Besides, RT‐qPCR analysis showed that RP11‐159K7.2 knockout significantly decreased DNMT3A levels compared with the negative group and no transfection group in LSCC cells (Figure 5G). In nude mice xenografts, the IHC results showed that DNMT3A was less expressed in RP11‐159K7.2 knockout tumour tissue than those in control (Figure 5H). In addition, we detected that DNMT3A level was decreased in LSCC cells transfected with DNMT3A siRNA, which could be rescued by miR‐206 inhibitors transfected together, as shown in Western blot. LSCC cells transfected with si‐DNMT3A had reduced proliferation and in vitro invasion ability as verified through colony formation and transwell assays. The inhibitive effect of si‐DNMT3A in malignant phenotypes of LSCC could be partially rescued by miR‐206 inhibitors cotransfection (Figure 6).

### 3.7 DNMT3A suppressed miR‐206 in a DNA methylation‐dependent manner

**FIGURE 7:**
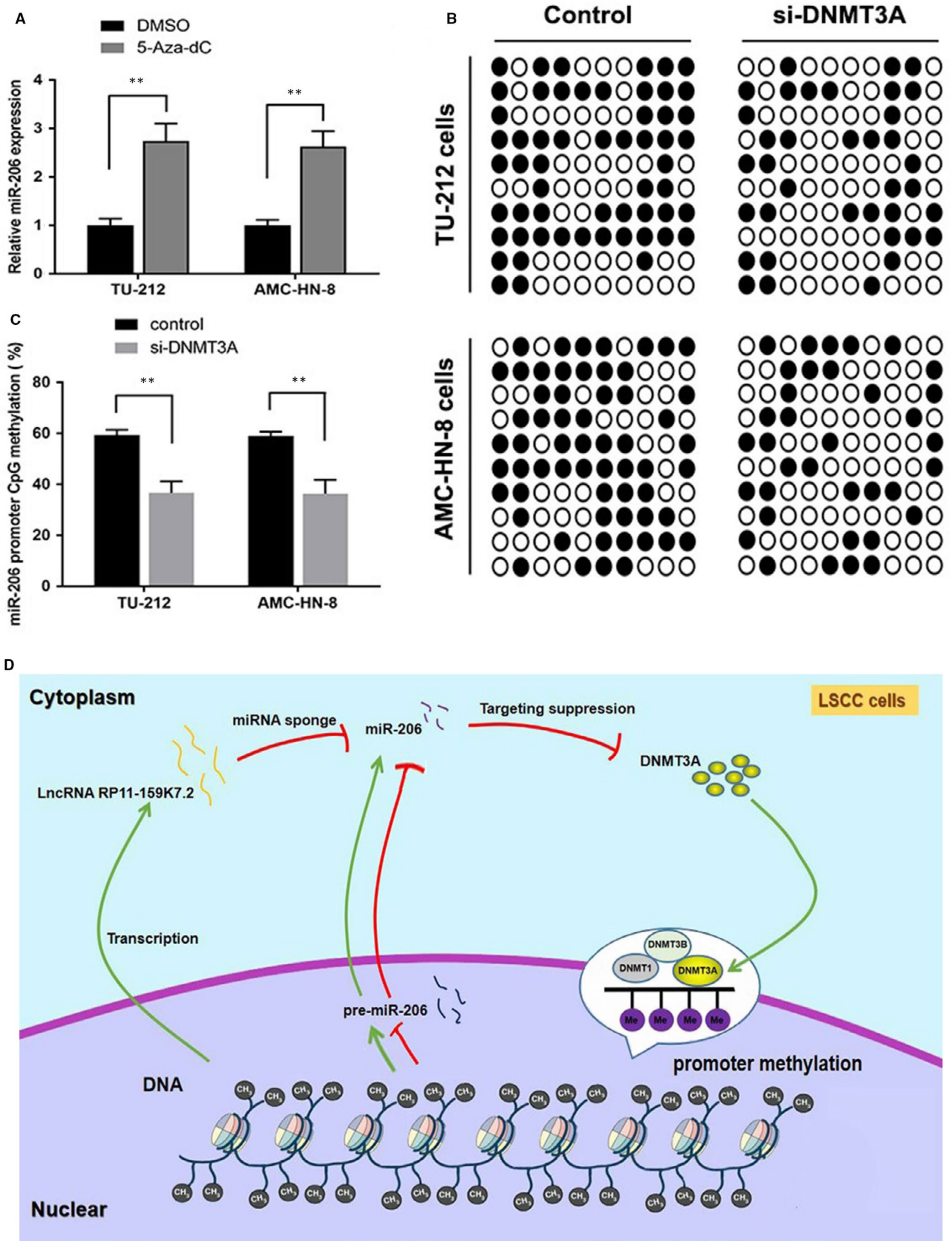
Negative feedback interaction between miR‐206 and DNMT3A. (A) miR‐206 expression levels in LSCC cells treated with 5‐Aza‐2’‐deoxycytidine. **P < .01. (B) Methylation profile in TU‐212 and AMC‐HN‐8 cell lines. The open and filled circles symbolized the unmethylated and methylated CpGs, respectively. (C) The methylation percentage of each cell line was shown. Error bars correspond to the mean ± SD (**P < .01). (D) Diagram summarizing the mechanism of action of RP11‐159K7.2/miR‐206/DNMT3A in LSCC cells. RP11‐159K7.2 competitively binds to miR‐206, which could directly combine with DNMT3A. DNMT3A also suppress miR‐206 through DNA methylation. The green arrows indicate promotive function, and red lines indicate suppressive function

Given that we often observe abnormal hypermethylation in carcinogenesis, that is critical for down‐regulating microRNAs, and that DNMT3A is a direct downstream target of miR‐206, we further proposed that there might be a negative feedback loop between miR‐206 and DNMT3A. To determine the effect of methylation on the expression levels of miR‐206, LSCC cells were treated with 5‐Aza‐dC. Compared with DMSO, treatment with 5‐Aza‐dC had a significant effect on the expression of miR‐206, indicating that miR‐206 expression in LSCC cells might be regulated by DNA methylation (**P < 0.01; Figure 7A). As shown in Figure 7B,C, we carried out BSP to evaluate the original methylation status of the identified CpG sites in LSCC cells. The percentage of miR‐206 promoter methylation in DNMT3A knockout cells in LSCC was 36.7% (TU‐212) and 36.3% (AMC‐HN‐8), respectively, which was significantly lower than in control groups. The above data indicated that the high methylation status of CpG sites might inhibit the expression of miR‐206 in LSCC. As summarized in Figure 7D, the obtained results confirmed our hypothesis, that is a negative feedback interaction between miR‐206 and DNMT3A.

## 4 DISCUSSION

Laryngeal cancer is one of the most common malignant tumours in the head and neck region. In 2015, there were 26 300 new cases and 14 500 deaths in China.^28^ According to the SEER Cancer Statistics Review, the 5‐year overall survival rate is approximately 60% in the United States.^29^, ^30^ Consequently, it is of utmost importance to find a reliable molecular marker and elucidate its molecular mechanisms.

Protein‐coding sequences account for only a tiny fraction (1%) of the genome, while the rest are associated with non‐coding RNA genes.^31^, ^32^ Several studies have reported that the lncRNA is essential for many critical cell regulatory processes, such as genomic imprinting, chromatin modification, transcriptional regulation and post‐transcriptional regulation.^33^, ^34^, ^35^ LncRNA and proteins form a complex regulatory network, through which they regulate cell proliferation, differentiation and apoptosis, and participate in many life activities.^36^, ^37^ Recently, the role of lncRNA in human cancers has aroused much attention, and increasing evidence has emphasized the clinical significance of lncRNA in neoplasms.^38^, ^39^, ^40^ Our previous studies suggested that the lncRNA HOTAIR, HOXA11‐AS and NEAT1 act as a proto‐oncogene in LSCC. Furthermore, the expression of lncRNA LET in laryngeal carcinoma cells is down‐regulated with clinical stage, and lymph node metastasis, and acts similarly to the tumour suppressor gene in laryngeal cancer.^17^, ^18^, ^19^, ^41^

So far, no studies have investigated the role of lncRNA RP11‐159K7.2 in cancers. To the best of our knowledge, this is the first study that examined the impact of lncRNA RP11‐159K7.2 on the development of LSCC. LncRNA RP11‐159K7.2 (Ensembl transcript ID: ENST00000508879.5) located on chromosome 5 is a 615 bp long lncRNA. In the present work, we discovered that the RP11‐159K7.2 was highly expressed in LSCC. We validated this finding in a cohort of 311‐paired tumorous and non‐tumorous tissues. We also found that the expression levels of RP11‐159K7.2 in LSCC tissues were associated with tumour classification, clinical stage, lymphatic invasion and recurrence.

CRISPR/Cas9 technology is a very efficient gene‐editing method for gene modification.^42^ Of note, CRISPR/Cas9 is the most investigated system applied in clinical trials of human cancer. To reveal the function of RP11‐159K7.2 in the proliferation and invasion of LSCC cells, both in vitro and in vivo experiments were carried out. Results showed that knockout of RP11‐159K7.2 by LentiCRISPR/Cas9 system, thus causing significant inhibition of cell proliferation and invasion. Furthermore, our results demonstrated that RP11‐159K7.2 knockout suppressed tumour growth in BALB/c mice xenografts. Besides, we also found that patients with low expression levels of RP11‐159K7.2 had an improved survival time, which suggested that RP11‐159K7.2 might be the molecular marker for predicting recurrence or evaluating prognosis in early LSCC.

In the present study, we found that down‐regulation of RP11‐159K7.2 can decrease the global DNA methylation levels of LSCC cells. DNMTs dysregulation is frequently reported in malignant tumours. Accordingly, we assumed that RP11‐159K7.2 might regulate the DNA methylation levels via DNA methylome. Through the method of bioinformatics analysis and luciferase reporter gene experiments, we found that miR‐206 can bind to RP11‐159K7.2 and regulate the target genes expression through ceRNA mechanism. miR‐206 plays a regulatory role in the process of DNA methylation of LSCC cells. Our previous study has shown that the expression of miR‐206 is decreased in LSCC and that the loss of miR‐206 is important for the proliferation, invasion and apoptosis of LSCC.^27^ As a key DNA methylation catalytic enzyme, DNMT3A has pivotal roles in de novo DNA methylation. DNMT3A was predicted to be a downstream target gene of miR‐206, which was further validated by experiments. In addition, DNMT3A has been reported to induce aberrant CpG site methylation in human cancer cells.^43^ Thus, we further investigated whether negative‐feedback regulations exist between miR‐206 and DNMT3A. We first detected an increased level of miR‐206 in LSCC cells treated with 5‐Aza‐2′‐deoxycytidine, which indicated that the expression of miR‐206 in LSCC cells was modified by DNA methylation. Then, BSP results demonstrated that down‐regulation of miR‐206 in LSCC cells was related to hypermethylation of CpG sites. Indeed, the decreased miR‐206 partially occurred due to DNMT3A‐mediated hypermethylation. In summary, our data indicate that RP11‐159K7.2 is overexpressed in LSCC, RP11‐159K7.2 can increases LSCC cell proliferation and invasion through its interaction with miR‐206 in a ceRNA manner, and miR‐206 binds DNMT3A through a complementary structure and is negatively regulated by RP11‐159K7.2. DNMT3A, as a methylase, is affected by RP11‐159K7.2 in LSCC, and its expression is increased, and miR‐206 is inhibited by increasing DNA methylation, thereby further reducing miR‐206 expression. These findings may be beneficial for the development of new treatment options for LSCC that target RP11‐159K7.2/miR‐206 and its downstream gene DNMT3A. Understanding the exact mechanism in LSCC will not only advance the knowledge of LSCC progression but also allow the development of particular and effective therapeutic strategies or biomarkers for predicting clinical outcomes. Therefore, CRISPR/Cas9‐mediated knockdown of the RP11‐159K7.2 gene may represent a more suitable and safer strategy for the management of LSCC than commonly used radiation or chemoradiation therapy. Of course, more animal model experiments are needed to ensure safety. Collectively, lncRNA RP11‐159K7.2 may function as a promising biomarker and therapeutic target for human LSCC treatment. Suppressing RP11‐159K7.2 can be a possible strategy to improve the clinical outcome of LSCC patients.

## CONFLICT OF INTEREST

The authors declare that they have no competing interests.

## AUTHORS' CONTRIBUTIONS

ML, LT and YS designed the study. XW, BY, QL and LY performed the cell experiments. XW, QJ, YL and BY performed animal experiments. JZ, PW and CS analysed the data. XW drafted the manuscript. YS revision the manuscript. All authors read and approved the final manuscript.

## Data Availability

The authors declare that the data in this article are available.
